# Clinical and pathological features analysis of invasive breast cancer with microcalcification

**DOI:** 10.1002/cam4.5848

**Published:** 2023-03-27

**Authors:** Yao Tian, Lu Zhao, Zhengwei Gui, Shiyang Liu, Chenguang Liu, Tianyao Yu, Lin Zhang

**Affiliations:** ^1^ Department of Thyroid and Breast Surgery Tongji Hospital of Tongji Medical College of Huazhong University of Science and Technology 1095 Jiefang Avenue, Qiaokou District Wuhan Hubei 430030 China

**Keywords:** HIF‐1α, immunohistochemistry, invasive breast cancer, microcalcification, OCN

## Abstract

**Purpose:**

Microcalcification (MC) is a valuable diagnostic indicator to detect invasive breast cancer (IBC). This study aimed to determine the clinicopathological features of IBC with MC and detect biomarkers related to the potential mechanism of the MC formation in IBC.

**Methods:**

Data from 364 patients with IBC were collected for the clinical characteristic analysis. The analysis of clinical data helped us to establish a predictive model of axillary node metastasis (ANM) before surgery. In addition, 49 tissue samples of IBC patients were collected to test the protein levels of osteocalcin (OCN) and hypoxia‐inducible factor‐1α (HIF‐1α) by immunohistochemistry.

**Results:**

Significant differences were observed in tumor size, age, ANM, HER2^+^, TNM stage, and mutant P53 between samples from IBC patients with MC and samples from IBC patients without MC. Younger age, a larger tumor size, a higher number of childbirths, and MC were independent predictors for ANM in IBC. HIF‐1α protein level was higher in tumor tissue than in normal tissue. High protein levels of OCN and HIF‐1α are related to the complication of MC in IBC. Of the patients that exhibited high HIF‐1α protein levels, the percentage of high OCN protein levels was larger in patients with ANM.

**Conclusion:**

Based on this study, we concluded that patients with MC had a comparatively poor prognosis. MC was an independent predictive factor associated with the risk of ANM. High protein levels of OCN and HIF‐1α were associated with MC and ANM, which were also related to poor prognosis. OCN and HIF‐1α had a positive correlation in IBC.

## Graphical abstract

MCs were an independent predictor for ANM risk. Our results provide a possibility for the prediction of ANM and prognosis in IBC and may offer novel therapeutic choices targeting calcification‐involved proteins.

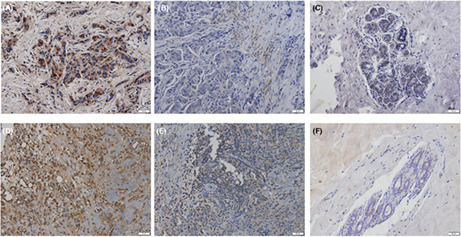

## 1 INTRODUCTION

Breast cancer is one of the female malignant cancers with high prevalence and lethality worldwide.^1^ The survival rate for breast cancer varies by the stage of breast cancer and therapies received.^2^ The application of various screenings and treatments has increased the detection rate of early breast cancers. Meanwhile, the survival rate has also increased. Microcalcification (MC) on mammography is the main malignant sign of breast cancer.^3^, ^4^, ^5^, ^6^ It is also a relatively specific and important marker for early breast cancer diagnosis.^7^ Broadly, MC refers to a calcification whose diameter is less than 1 mm. Breast MCs consist of either calcium oxalate crystals, which often indicate benign breast conditions, or hydroxyapatite crystals, related to both malignant and benign breast diseases.^8^ In the breast cancer risk stratifications and prognostic analyses, the mammographic performance, and components of MCs are sufficiently taken into consideration.^9^

Currently, accumulating evidence shows that osteoblast‐like cells emerge in breast cancer with MCs, suggesting that calcification is an active process in breast cancer tissue.^10^ Similarly, growing evidence suggests that MCs play a significant role in breast cancer initiation and progression.^7^ Several hypotheses suggest that MC formation is a cellular process in which the phenotype of breast cancer cells transforms to a mineralized type.^11^, ^12^ Other hypotheses support the view that MC in invasive breast cancer (IBC) consists of cell necrosis and debris.^13^, ^14^, ^15^ MCs of IBC mostly consist of hydroxyapatite.^16^, ^17^ The mechanism of MC formation in IBC remains controversial Recently studies have shown that pathological MCs may resemble the ectopic bone formation process.^18^, ^19^, ^20^

Therefore, in this research, we retrospectively analyzed our database to assess the association between MC and clinicopathological characteristics in IBC. We incorporated tumor size, axillary node metastasis (ANM), age, tumor molecular markers, clinical stage, and the number of births. To discover biomarkers associated with the potential mechanism of MC formation, we examined the expression of osteocalcin (OCN) and hypoxia‐inducible factor‐1α (HIF‐1α), which are important markers of osteogenic approaches in MC tissue, in the development of IBCs with or without MCs.^21^, ^22^, ^23^

## 2 METHODS

### 2.1 Study population

The present survey was permitted by the Institutional Review Board of Tongji Hospital (Wuhan, China) (TJ‐IRB20221137). The retrospective analysis included female patients who were confirmed to have unilateral IBC from 2018 to 2019. Inclusion required adults aged 18 years or older, primary unilateral invasive IBC, and preoperative diagnostic mammography. Patients diagnosed with only carcinoma in situ were eliminated. Female patients who underwent medical breast cancer treatment before surgery or had a history of malignant neoplasms including breast cancer were excluded. Finally, for data integrity and follow‐up, 364 patients with IBC in recent years were selected for retrospective analysis.

### 2.2 Tissue samples

All participants signed an informed consent form. A total of 27 samples of IBC with MC group and IBC without MC group (22 cases total) were gathered from patients who received surgical treatment at Tongji Hospital from March to December 2020. Until the end of the experiment, we could only collect 49 tumor samples for immunohistochemistry. Most parts of the samples were excluded finally for combining with ductal carcinoma in situ. In the study, all samples were fixed in 4% paraformaldehyde and then sectioned into 4 μm slices after embedding in paraffin. All tissue specimens were gained from the patients prior to any medical therapy. To obtain the sample tissues with MC, we prospectively collected fresh tissues during the surgery. This study followed the Helsinski Declaration.

### 2.3 Immunohistochemistry techniques

The expression levels of OCN and HIF‐1α were immunohistochemically detected in paraffin‐embedded tissue samples. Slides (4 μm thick) were incubated at 60°C overnight. The slides were deparaffinized in xylene. We utilized 3% hydrogen peroxide to block endogenous peroxidase activity at indoor temperature for 10 min and washed the slides with phosphate‐buffered saline. Then the slides were blocked with goat serum for 20 min at room temperature. The specimens were incubated with a primary antibody against HIF‐1α (1:50; rabbit polyclonal; 20,960‐1‐AP; Proteintech), or OCN (1:50; rabbit polyclonal; 23,418‐1‐AP; Proteintech), and held at 4°C overnight. Following this, horseradish peroxidase‐conjugated goat anti‐rabbit IgG was added as a secondary antibody at room temperature for 30 min. Then, the slides were treated with DAB (diaminobenzidine) for 5–10 min, counterstained with hematoxylin, dehydrated in alcohol and xylene, and then mounted. Finally, the sections were sealed with neutral gum.

### 2.4 Evaluation of immunohistochemical staining

The ImageJ software was used for immunohistochemical analysis. The staining of OCN and HIF‐1α expression was evaluated by semiquantitative grading as follows: The percentage and intensity of positive stained cells was evaluated in randomly selected high‐power views (200×) of each sample using the ImageJ software. The extent of staining was scored based on the percentages of positive cells: 0 (0–5%), 1 (6–25%), 2 (26–50%), 3 (51–75%), and 4 (>75%). The intensity of staining was determined as follows: score 0 for negative, score 1 for low positive, score 2 for positive, and score 3 for high positive. The outcome of the final staining score was defined as the multiplication of the percentage and intensity scores: 0 (negative), I (1–4), II (5–8), and III (9–12). For further statistical analysis, we defined a low‐protein‐expression group of HIF‐1α or OCN for scores of 0 or I, while scores of II or III severed as a high‐protein‐expression group of HIF‐1α or OCN.

### 2.5 Statistical analysis

Continuous variables that obeyed a normal distribution were analyzed with Student's *t*‐test (two‐tailed) and expressed as the means with standard deviation. The Mann–Whitney *U*‐test was performed when continuous variables were nonnormally distributed, which are presented as medians with interquartile range. Categorical variables, shown as proportions, were analyzed by the chi‐squared test. The Spearman rank was used to detect the correlation of OCN and HIF‐1α. In the research, *p* < 0.05 was regarded to be statistically significant. SPSS 24.0 statistical software was used for the statistical analysis.

We selected candidate variables with univariate logistic regression analysis. Then, multivariable regression was conducted for candidate variables with *p* < 0.2 in the univariate analysis. A nomogram predicting ANM risk was established based on variables with *p* < 0.05 in the multivariate analysis using the R package rms. The predictive capabilities were evaluated by drawing the calibration curve and the receiver operating characteristic (ROC) curve.

## 3 RESULTS

### 3.1 Correlation of MC with clinical characteristics

A total of 364 IBC patients who received definitive surgery at our institution were enrolled in the research. All patients were female. In the cohort, the patients' median age was 50 years. In the study, 51.6% of the female patients were postmenopausal and 48.4% of the female patients were premenopausal. A total of 15.1% of the participants received breast‐conserving surgery, while 82.7% of them underwent mastectomy. Only 2.20% of patients had mastectomy with reconstruction.

Among the 364 participants, 180 cases (49.45%) were confirmed to have MCs by mammography, and 184 cases (50.55%) did not display MCs on the images (Table 1). All of the variables were calculated preoperatively, from imaging or from biopsy except for ANM and TNM stage. Age refers to the age of confirmed diagnosis by pathology. A *t*‐test showed that age significantly differed between the above two groups (49.10 vs. 51.77, *p* = 0.013). According to Mann–Whitney *U*‐test analysis, the difference in tumor size was significant (*p* < 0.001) between the two groups. The chi‐squared test analysis results hinted that TNM stage (*p* < 0.001), axillary node status (ANM) (*p* = 0.005), HER2 amplification status (*p* < 0.001), and mutant P53 (*p* = 0.015) were both significantly different between the IBC with MC and IBC without MC groups. Nevertheless, menstrual status (*p* = 0.092), surgical mode (*p* = 0.123), PR (*p* = 0.211), ER (*p* = 0.237), and Ki‐67(*p* = 0.111) displayed no significant differences.

**TABLE 1:** Clinical characteristics in different subgroups of IBC.

Characteristic	IBC with microcalcification (*n* = 180)	IBC without microcalcification (*n* = 184)	*p*
Age, years			**0.013**
Mean ± SD	49.10 ± 10.98	51.77 ± 9.29	
Menstrual status, *n* (%)			0.092
Premenopausal	101 (56.10)	87 (47.30)	
Postmenopausal	79 (43.90)	97 (52.70)	
Number of childbirths	1 (1,2)	1 (1,2)	0.351
Tumor size, cm			**<0.001**
Median (IQR)	2.50 (2.00, 3.00)	2.00 (1.50, 2.50)	
TNM stage, *n* (%)			**<0.001**
I	25 (13.89)	63 (34.24)	
II	112 (62.22)	94 (51.09)	
III	43 (23.89)	27 (14.67)	
ANM, *n* (%)			**0.005**
No	83 (46.11)	112 (60.87)	
Yes	97 (53.89)	72 (39.13)	
IHC, *n* (%)			
ER^+^	125 (69.44)	138 (75.00)	0.237
PR^+^	110 (61.11)	124 (67.39)	0.211
HER2^+^	69 (38.33)	35 (19.02)	**<0.001**
TNBC	17 (9.44)	34 (18.48)	**<0.001**
Mutant P53, *n* (%)	81 (45.00)	56 (30.43)	**0.015**
Ki‐67, *n* (%)			0.111
LI <20%	37 (20.56)	51 (27.72)	
LI ≥20%	143 (79.44)	133 (72.28)	
Surgery, *n* (%)			0.123
BCT	22 (12.22)	33 (17.93)	
Mastectomy	152 (84.45)	149 (80.98)	
Mastectomy+BR	6 (3.33)	2 (1.09)	

*Note*: Bold value indicates statistical significance.

Abbreviations: −, negative; +, positive; ANM, axillary node metastasis; BCT, breast‐conserving therapy; BR, breast reconstruction; ER, estrogen receptor; HER2, human epidermal growth factor receptor 2; IBC, invasive breast cancer; IHC, immunohistochemisty; IQR, interquartile range; LI, label index; PR, progesterone receptor; SD, standard deviation; TNBC, triple‐negative breast cancer.

**FIGURE 1:**
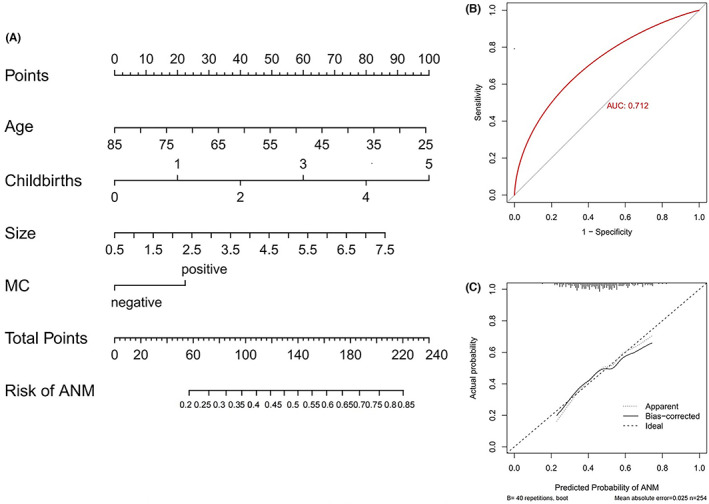
Construction of the axillary node metastasis risk model. (A) Nomogram to predict the rate of axillary node metastasis (ANM) in invasive breast cancer patients before surgery. (B) Receiver operating characteristic (ROC) curves of the nomogram. The nomogram had a good discriminative performance with area under ROC curve of 0.712. (C) Calibration curve comparing predicted and actual axillary node‐metastasis‐risk probabilities.

Based on the available data listed in Table 1, these 364 patients were grouped into a training set and testing set at a ratio of 7:3 to build a predictive model of ANM risk. The variables included in our model were collected before the surgery. Variables with *p* < 0.2 were age (*p* = 0.031), number of childbirths (*p* = 0.108), tumor size (*p* = 0.008), ER status (*p* = 0.167), PR status (*p* = 0.107), MC (*p* = 0.005), and Ki‐67 (*p* = 0.106). The above variables were incorporated into the multivariable logistic regression model to select the candidate predictors for the risk of ANM preoperatively. On the basis of multivariate analysis, we found that younger age (0.975 [0.953–0.998]), larger tumor size (1.319 [1.037–1.677]), multiparity (1.461 [1.070–1.996]), and tumors with MC (1.586 [1.014–2.480]) were independent predictors for the risk of ANM (Table 2). The independent predictors were utilized to build an estimation nomogram for the predictive model of ANM risk before surgery, as shown in Figure 1. The nomogram showed satisfactory precision to predict ANM, with a C‐index of 0.712. The ROC curve also showed good diagnostic accuracy (Figure 1B). The calibration plot showed a good coherence between the bias‐corrected prediction line and the ideal reference line (Figure 1C).

**TABLE 2:** Multivariate logistic regression analysis of ANM based on data before surgery.

Variables	*p*	OR (95% CI)
Age (per 1‐year increase)	**0.037**	0.975 (0.953–0.998)
Tumor size (per 0.1 cm increase)	**0.024**	1.319 (1.037–1.677)
Number of childbirths (per 1 increase)	**0.017**	1.461 (1.070–1.996)
Microcalcification (present vs. absent)	**0.043**	1.586 (1.014–2.480)
ER (negative vs. positive)	0.375	1.429 (0.650–3.144)
PR (negative vs. positive)	0.302	1.453 (0.714–2.957)
Ki67 (per 1% increase)	0.051	1.013 (1.000–1.026)

*Note*: Bold value indicates statistical significance.

Abbreviations: ER, estrogen receptor; PR, progesterone receptor.

### 3.2 Immunohistochemistry for OCN and HIF‐1α expression in IBC

**FIGURE 2:**
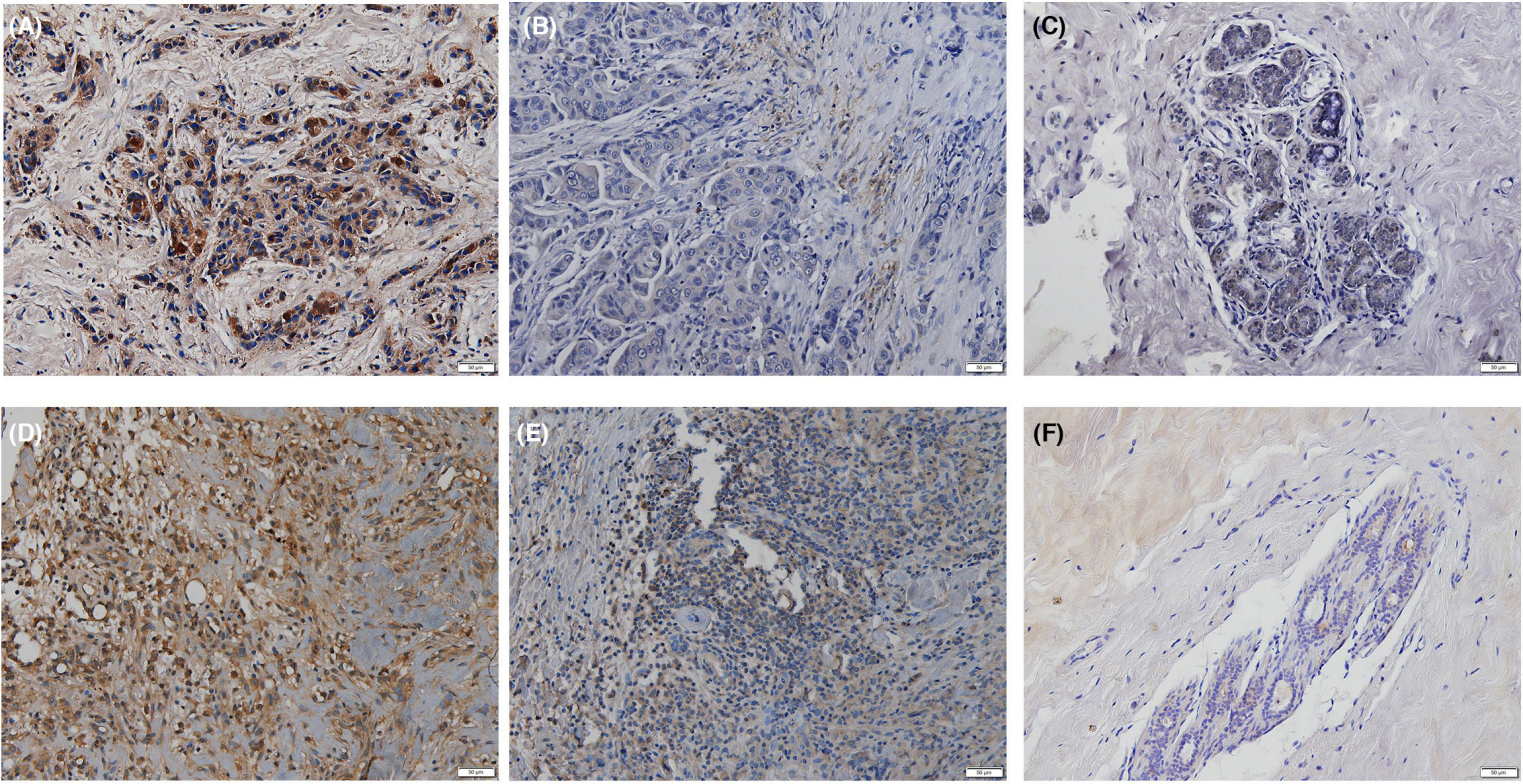
Immunohistochemical analysis of hypoxia‐inducible factor‐1α (HIF‐1α) and osteocalcin (OCN) in invasive breast cancer (IBC). (A, D) Representative images of HIF‐1α (A) and OCN (D) in IBC with microcalcification. (B, E). Representative images of HIF‐1α (B) and OCN (E) in IBC without microcalcification. (C, F) Representative images of HIF‐1α (C) and OCN (F) in normal tissue; ×200).

**FIGURE 3:**
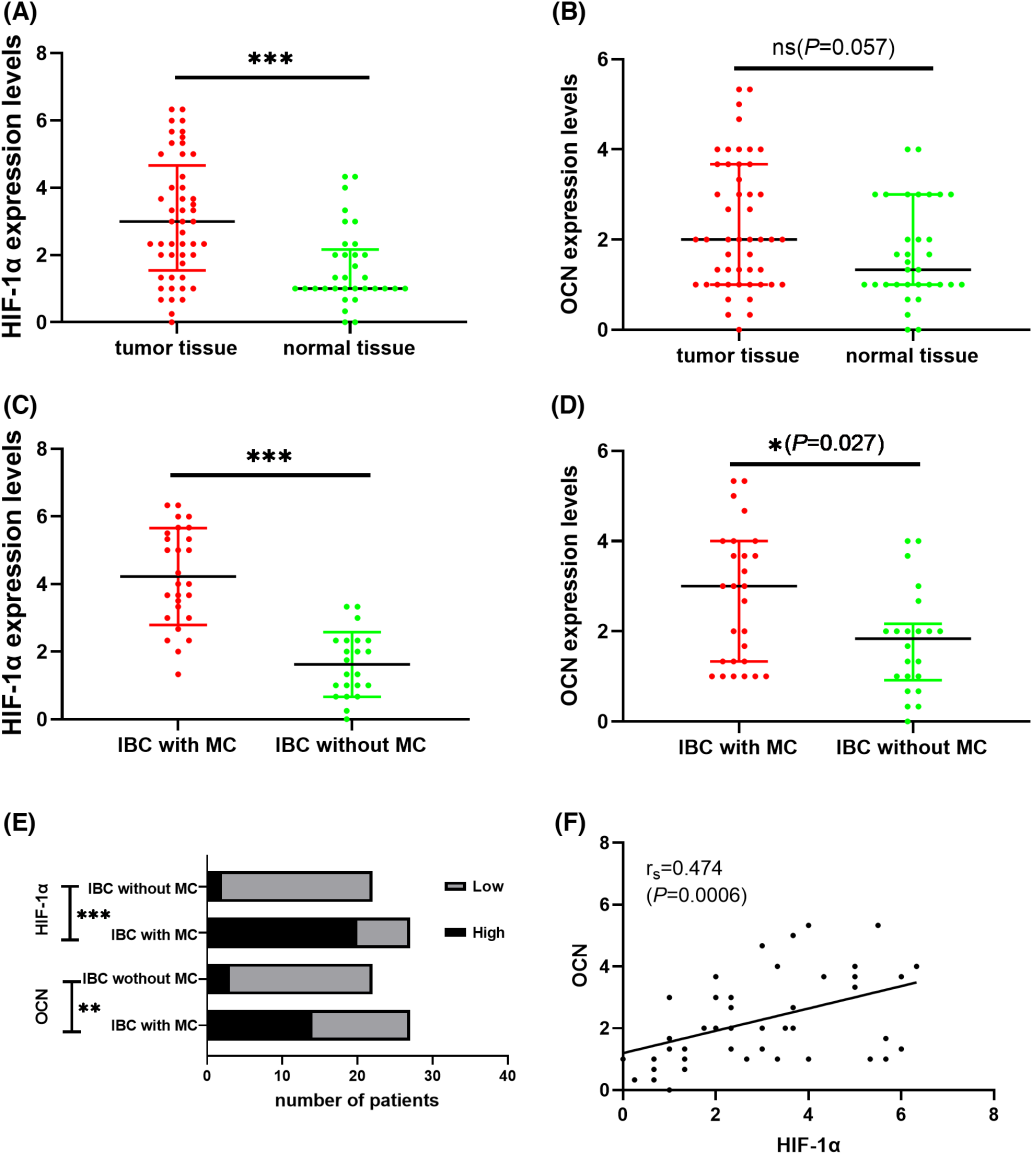
Protein levels of osteocalcin and hypoxia‐inducible factor‐1α.

First, 49 specimens of IBC tumor tissues and 33 samples of normal tissues were tested for the expression of OCN and HIF‐1α. Positive staining of OCN and HIF‐1α was discovered primarily in the cytoplasm (Figure 2). In comparison with normal tissue, the protein level of HIF‐1α was significantly higher in tumors (*p* < 0.001) (Figure 3A). However, the OCN level was not found a statistical difference (*p* = 0.057) (Figure 3B) between normal tissues and tumor tissues. Among the tumor tissues, the levels of OCN (*p* = 0.027) and HIF‐1α (*p* < 0.001) both showed significant differences between the IBC with MC and the non‐MC groups (Figure 3C,D). The differences in OCN (*p* = 0.005) and HIF‐1α (*p* < 0.001) protein high expression between the IBC with MC and IBC without MC groups were statistically significant (Figure 3E). High HIF‐1α levels were detected in 20 (90.91%) IBCs with MC and in 2 (9.09%) IBCs without MC. Consistently, high OCN levels were also found in 14 (82.35%) IBCs with MC compared with 3 (17.65%) IBCs in the non‐MC group.

### 3.3 Correlations of OCN and HIF‐1α levels with clinicopathological characteristics

Regarding the clinical data, the age distribution did not correlate with OCN (*p* = 0.606) or HIF‐1α (*p* = 0.687) level. Moreover, tumor size was not related to high levels of HIF‐1α in IBC tissues (*p* = 0.461) but was associated with high OCN levels (*p* = 0.028). Both high OCN (*p* = 0.005) and HIF‐1α (*p* < 0.001) protein levels were significantly related to the complication of the MC in IBC. No significant differences were found for the remaining variables (Table 3).

**TABLE 3:** Association of clinicopathological parameters with OCN or HIA‐1α expression.

Characteristic	IBC total, *n* (%)	High HIF‐1α	*p*	High OCN	*p*
	49 (100.00)	22 (44.90)		17 (34.69)	
Age, years Median (IQR)	52 (41.00, 58.00)	51 (37.00,58.25)	0.687	50 (39.00, 58.00)	0.606
Menstrual status, *n* (%)			0.698		0.224
Premenopausal	26 (53.06)	11 (50.00)		10 (58.82)	
Postmenopausal	23 (46.94)	11 (50.00)		7 (41.18)	
Tumor size, cm Median (IQR)	2.50 (2.00, 3.20)	2.60 (2.00, 3.25)	0.461	3.00 (2.15, 4.00)	**0.028**
TNM stage, *n* (%)			0.796		0.732
I	10 (20.41)	5 (22.73)		3 (17.65)	
II	28 (57.14)	13 (59.09)		11 (64.71)	
III	11 (22.45)	4 (18.18)		3 (17.65)	
ANM, *n* (%)			0.482		0.108
No	25 (51.02)	10 (45.45)		6 (35.29)	
Yes	24 (48.98)	12 (54.55)		11 (64.71)	
IHC, *n* (%)					
ER^+^	35 (71.43)	18 (81.82)	0.146	14 (82.35)	0.217
PR^+^	30 (61.22)	16 (72.73)	0.136	13 (76.47)	0.110
HER2^+^	9 (18.37)	5 (60.00)	0.477	4 (23.53)	0.496
P53, *n* (%)			0.082		0.638
Wild‐type	31 (63.27)	11 (50.00)		10 (58.82)	
Mutant	18 (36.73)	11 (50.00)		7 (41.18)	
Ki‐67, *n* (%)			0.074		0.060
LI <20%	10 (20.41)	7 (31.82)		6 (35.29)	
LI ≥20%	39 (79.59)	15 (68.18)		11 (64.71)	
Microcalcification			**<0.001**		**0.005**
No	22 (44.90)	2 (9.09)		3 (17.65)	
Yes	27 (55.10)	20 (90.91)		14 (82.35)	

*Note*: Bold value indicates statistical significance.

Abbreviations: −, negative; +, positive; ANM, axillary node metastasis; BCT, breast‐conserving therapy; BR, breast reconstruction; ER, estrogen receptor; HER2, human epidermal growth factor receptor 2; IBC, invasive breast cancer; IHC, immunohistochemisty; IQR, interquartile range; LI, label index; PR, progesterone receptor; SD, standard deviation; TNBC, triple‐negative breast cancer.

We divided the 49 patients with IBC into high‐ and low‐ HIF‐1α protein groups, and the association of OCN protein level with clinical characteristics was investigated in these two groups (Table 4). Among the patients with high HIF‐1α levels, the proportion of high OCN protein was higher in patients with ANM (*p* = 0.035), but low OCN levels were more often present in IBC patients with a Ki67 index of more than 20% (*p* = 0.045). The remaining variables had no statistically significant differences in this group. In the patients with low HIF‐1α protein levels, patients with high OCN protein levels were more accessible to be complicated with MC (*p* = 0.002). Spearman rank test was conducted to discover the association between OCN and HIF‐1α in tumor tissues. As displayed in Figure 3F, a significantly different positive correlation was observed between OCN and HIF‐1α (*r*_s_ = 0.474, *p* < 0.001).

**TABLE 4:** Comparison of clinicopathologic parameters by level of OCN IHC in IBC patients with or without HIF‐1α protein over‐expression.

Characteristic	High HIF‐1α (*n* = 22)			Low HIF‐1α (*n* = 27)		
	High OCN (*n* = 12)	Low OCN (*n* = 10)	*p*	High OCN (*n* = 5)	Low OCN (*n* = 22)	*p*
Age, years Median (IQR)	51 (34.00,62.25)	51 (39.25,58.00)	0.843	47 (42.50,54.00)	54 (46.25,58.25)	0.317
Menstrual status, *n* (%)			1.000			0.076
Premenopausal	6 (50.00)	5 (50.00)		4 (80.00)	8 (36.36)	
Postmenopausal	6 (50.00)	5 (50.00)		1 (20.00)	14 (63.64)	
Tumor size, cm Median (IQR)	3.00 (2.08, 4.00)	2.50 (2.00, 2.73)	0.124	2.80 (2.20, 3.10)	2.45 (2.00, 3.50)	0.731
TNM stage, *n* (%)			0.576			0.323
I	2 (16.67)	3 (30.00)		1 (20.00)	4 (18.18)	
II	7 (58.33)	6 (60.00)		4 (80.00)	11 (50.00)	
III	3 (25.00)	1 (10.00)		0 (0.00)	7 (31.82)	
ANM, *n* (%)			**0.035**			0.825
No	3 (25.00)	7 (16.67)		3 (60.00)	12 (54.55)	
Yes	9 (75.00)	3 (30.00)		2 (40.00)	10 (45.45)	
IHC, *n* (%)						
ER^+^	10 (83.33)	8 (80.00)	0.840	4(80.00)	13 (59.09)	0.382
PR^+^	9 (5.00)	7 (70.00)	0.793	4 (80.00)	9 (40.91)	0.135
HER2^+^	2 (16.67)	3 (30.00)	0.781	3 (60.00)	1 (4.55)	0.718
P53, *n* (%)			0.087			0.054
Wild‐type	8(66.67)	3 (30.00)		2 (40.00)	18 (81.82)	
Mutant	4 (33.33)	7 (70.00)		3 (60.00)	4 (18.18)	
Ki67, *n* (%)			**0.045**			0.381
LI <20%	6 (50.00)	1 (10.00)		0 (0.00)	3 (13.64)	
LI ≥20%	6 (50.00)	9 (90.00)		5 (100.00)	19 (86.36)	
Microcalcification			0.176			**0.002**
No	2 (16.67)	0 (0.00)		1 (20.00)	19 (86.36)	
Yes	10 (83.33)	10 (100.00)		4 (80.00)	3 (13.64)	

*Note*: Bold value indicates statistical significance.

Abbreviations: −, negative; +, positive; ANM, axillary node metastasis; BCT, breast‐conserving therapy; BR, breast reconstruction; ER, estrogen receptor; HER2, human epidermal growth factor receptor 2; IBC, invasive breast cancer; IHC, immunohistochemisty; IQR, interquartile range; LI, label index; PR, progesterone receptor; SD, standard deviation; TNBC, triple‐negative breast cancer.

## 4 DISCUSSION

IBC is a clinically heterogeneous disease that deviates remarkably from one another in regard to morphology, subtypes with distinct clinicopathological characteristics, sensitivity to therapeutic regimens, and prognosis. MC was observed alone or in correlation with masses on radiology as a malignant sign of breast carcinoma.^24^ Mammography, the most sensitive method for the examination of MC, has been applied in clinical practice for many years and is an essential method in the early diagnosis of breast cancer. MCs in breast cancer primarily exhibit distribution along the duct, clustered distribution, and segmented and diffuse distribution. Morphologically, the MC characteristics of breast cancer include amorphous, fine pleomorphic, and fine‐linear branching.^25^, ^26^ The prevalence of MC in IBC patients has been reported to differ domestically and internationally, accounting for 31.95%–45.70% of IBC patients.^4^, ^27^, ^28^ In this study, the incidence of MC in IBC patients was 49.45%, similar to that reported in the literature.

In this investigation, we inspected the connection between mammographic MCs and clinicopathological features among patients with IBC. Previous studies have reported correlations between calcifications and unfavorable prognostic characteristics and worse survival.^29^, ^30^, ^31^ Tsau et al. found that MC increased the mortality hazard ratio by 3.47‐fold in a study of 498 participants diagnosed with IBC after adjusting for other prognostic features.^32^ Analogously, a recent study showed that MC was an independent prognostic factor for patients with breast cancer.^33^ However, an investigation of 470 women with IBC detected by screening concluded that mammographic MC had no influence on breast cancer‐specific survival.^34^ Many researches have also revealed correlations between MC and the risk of recurrence in breast cancer. A study reported that patients with calcifications showed a markedly increased local recurrence rate.^35^ However, another research of 937 cases of IBC revealed no significant difference between MC and recurrence.^30^ Thus, the cohort of 364 IBC patients in our study was grouped on the basis of MC (presence or absence), aiming to accurately assess the relationship between clinicopathological features and MC.

Our research showed that larger tumors were more common in the MC group (*p* < 0.001). The patients in the MC group tended to be younger (*p* = 0.013). The percentages of patients with ANM (53.89% vs. 39.13%, *p* = 0.005), HER2 overexpression (*p* < 0.001), and P53 mutation (*p* = 0.015) were higher in the MC group. Zhang et al.^36^ reported that the proportion of ANM was 35% in their calcification group, higher than the 27.9% value in the control group. Cen et al.^37^ also verified a remarkably higher rate of ANM in breast cancer patients with calcification than in patients without calcification in a total of 419 patients. However, our study showed that several variables, including ER, PR, Ki‐67, and menopausal status, were not related to MC in IBC. However, these results apparently differed from those of previous researches which reported associations between calcifications and hormone receptor‐negative tumors.^38^, ^39^ Conversely, a study including 8472 patients with IBC revealed that calcifications were associated with small tumor size and hormone receptor positivity.^40^ Thus, more extensive and in‐depth research is necessary. In our study, patients with MC often showed larger tumors and higher risks of ANM, which indicated a higher malignant grade of carcinomas. The multivariable regression also showed that MCs were an independent predictor for ANM. As shown in our data, the surgical methods for IBC did not differ in the with or without MC groups. ANM is one of the most significant predictor of overall survival in patients with IBC.^41^, ^42^, ^43^ However, patients with MC were more likely to develop ANM, indicating a relatively poor prognosis. Therefore, the choice of surgical option should be more inclined to mastectomy. Nevertheless, the above conclusions all warrant further research.

In solid tumors, hypoxia is a common phenomenon and has been increasingly acknowledged to play an important role in tumor progression.^44^ HIFs and HIF‐1 signal regulatory pathways are prominently valuable in the hypoxic response.^45^ HIF‐1α has been regarded as a promoter for tumor development and tumorigenesis.^46^ HIF‐1α, as an important regulator of cell adjustment to hypoxia, is frequently upregulated in cancers dhypoxia in tumors or the activation of various oncogenic pathways.^47^ Several studies revealed that the activation of the HIF‐1α related pathway can contribute to the induction of osteogenic differentiation.^48^, ^49^ OCN is a late osteoblast differentiation marker that is highly expressed during the process of osteogenic differentiation.^50^, ^51^ However, most of the studies have focused on the serum OCN concentration, which is an indicator of bone metastasis.^43^, ^54^, ^55^

Nevertheless, studies have not tested the expression of OCN and HIF‐1α under equal conditions methodically and concurrently assessed the association of their protein levels with clinicopathological characteristics in IBC. In the present survey, we concluded that the level of HIF‐1α was higher in tumor tissues (*p* < 0.001), while OCN did not show a similar trend (*p* = 0.057). However, this difference was statistically significant between the IBCs with or without MC, both for HIF‐1α and OCN. The above results indicate that the higher levels of OCN and HIF‐1α were more often present in IBCs with MC.

The association of high OCN and HIF‐1α protein levels with various clinicopathological characteristics was also evaluated. We recognized that OCN and HIF‐1α levels were not related to age, menstrual status, TNM stage, ANM, ER, PR, HER2, or Ki‐67. Yet, high OCN levels were significantly related to tumor size (*p* = 0.028) and MC (*p* = 0.005). High HIF‐1α levels were also associated with MC (*p* < 0.001). Significantly, this survey first elaborated a positive correlation between OCN and HIF‐1α levels in IBC (*p* < 0.001). From these results, we hypothesized that the higher level of OCN showed that the formation of breast cancer MCs was associated with osteogenic differentiation and that HIF‐1α was involved in the regulation of this process. A similar exploration of the correlation of HIF‐1α and OCN has been conducted in vascular smooth muscle cells, which concluded that active OCN signaling could induce the activation of HIF‐1α, which finally leads to vascular calcification.^21^ The potential mechanisms of this correlation warrant exploration. Nevertheless, the present research primarily concentrated on tissues. More comprehensive mechanistic studies at the cellular and molecular levels are required in the future.

There are several limitations in our research. First, this is a single‐center retrospective research, which inevitably gives rise to unintentional selection bias. Second, our strict entry criteria led to a relatively small number of participants, thus, the predictive power of our nomogram is limited. Third, in order to obtain the immunohistochemical results of calcified IBC tissues, the samples complicated with carcinoma in situ were excluded. As a result, we finally gained a relatively small number of tissues samples, which may affect the accuracy of the final result.

## 5 CONCLUSION

In summary, MCs were an independent predictor for ANM risk. The incorporation of MC yielded a more accurate preoperative clinical predictive model for ANM. To the best of our knowledge, this study is the first to expound the positive association between OCN and HIF‐1α in IBC. High expression levels of OCN and HIF‐1α were related to MC and ANM. Therefore, our results provide a possibility for the prediction of ANM and prognosis in IBC and may offer novel therapeutic choices targeting calcification‐involved proteins. However, the potential mechanisms still require further exploration. Prospective researches in larger samples of patients will be indispensable to determine the utility of these two molecules as biomarkers for tumor diagnosis, prognosis, and therapy in IBC.

## AUTHOR CONTRIBUTIONS

**Yao Tian:** Data curation (equal); investigation (equal); writing – original draft (equal). **Lu Zhao:** Formal analysis (equal); supervision (equal). **Zhengwei Gui:** Resources (equal). **shiyang liu:** Writing – review and editing (equal). **Chenguang Liu:** Investigation (equal). **tianyao yu:** Investigation (equal). **Lin Zhang:** Conceptualization (equal); funding acquisition (equal); writing – review and editing (equal).

## FUNDING INFORMATION

This study was partially funded by the National Natural Science Foundation of China (21834002). The funding source had no role in the study other than financial support.

## CONFLICT OF INTEREST STATEMENT

All authors declare no competing interest.

## ETHICS STATEMENT

All procedures performed in studies involving human participants were in accordance with the Institutional Review Board of Tongji Hospital (Wuhan, China) and with the 1964 Declaration of Helsinki and its later amendments or comparable ethical standards.

## Supporting information


**Figure S1.** Study population and tissue samples enrolment in the research. (A) Study population enrolment; (B) Tissue samples enrolment

**Table S1.** Univariate logistic regression analysis of ANM based on data before surgery.

## Data Availability

Data Availability Statement: Data are available on request.
