# Supplementary figures and images for: Clinical and pathological features analysis of invasive breast cancer with microcalcification
Source: Cancer Med. 2023 Mar 27;12(10):11351–62. doi: 10.1002/cam4.5848 (PMC10242326; doi:10.1002/cam4.5848)

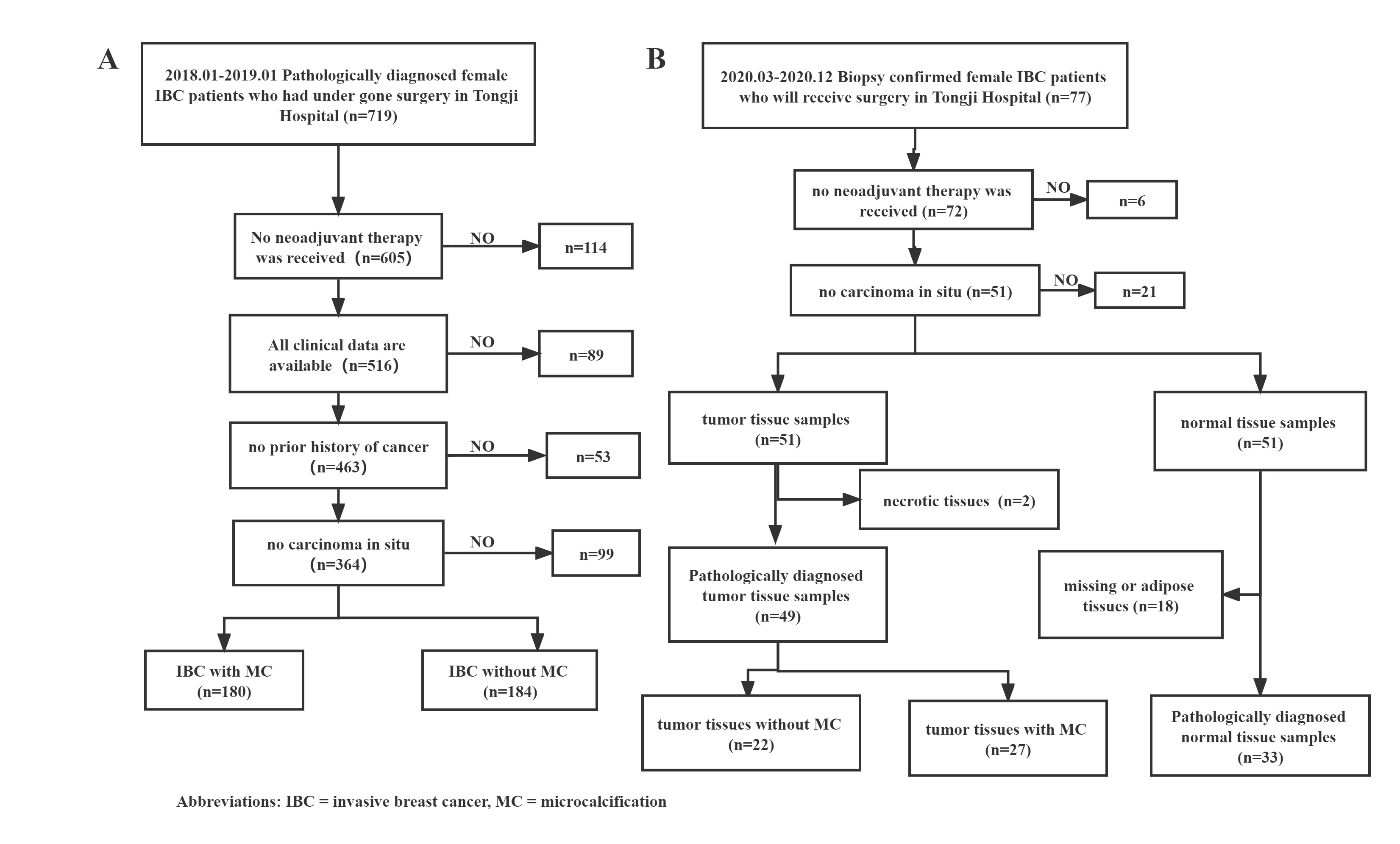

Supplement: Supplementary file 1 — Figure S1. Study population and tissue samples enrolment in the research. (A) Study population enrolment; (B) Tissue samples enrolment [file CAM4-12-11351-s002.jpg]
