# Supplementary material for: Clinical and pathological features analysis of invasive breast cancer with microcalcification
Source: Cancer Med. 2023 Mar 27;12(10):11351–62. doi: 10.1002/cam4.5848 (PMC10242326; doi:10.1002/cam4.5848)
Supplement: Supplementary file 2 — Table S1. Univariate logistic regression analysis of ANM based on data before surgery. [file CAM4-12-11351-s001.docx]

**Table S1.** Univariate logistic regression analysis of ANM based on data before surgery.

| **Variables** | **β** | ***P*** | **OR (95%CI)** |
| --- | --- | --- | --- |
| **age (per 1-year increase)** | -0.023 | **0.031** | 0.978 (0.958-0.998) |
| **tumor size (per 0.1cm increase)** | 0.295 | **0.008** | 1.343 (1.079-1.673) |
| **number of childbirths (per 1 increase)** | 0.229 | **0.108** | 1.257 (0.951-1.662) |
| **microcalcification (present vs. absent)** | -0.598 | **0.005** | 0.550 (0.363-0.834) |
| **ER (negative vs. positive)** | -0.328 | **0.167** | 0.721 (0.453-1.147) |
| **PR (negative vs. positive)** | -0.356 | **0.107** | 0.700 (0.454-1.080) |
| HER2 (negative vs. positive) | 0.070 | 0.765 | 1.072 (0.679-1.693) |
| **Ki67 (per 1% increase)** | 0.009 | **0.106** | 1.009 (0.998-1.020) |
| Subtype   Luminal B vs. Luminal A | 0.011 | 0.973 | 1.011 (0.548-1.863) |
| TNBC vs. Luminal A | -0.221 | 0.394 | 0.801 (0.482-1.333) |
| HER2^+^ vs. Luminal A | -0.245 | 0.481 | 1.278 (0.646-2.528) |

Abbreviations: ER = estrogen receptor, PR = progesterone receptor, HER2=Human epidermal growth factor receptor 2, TNBC=triple-negative breast cancer, CI=confidence interval

Bold value are variables with *P*<0.2 which are candidate variables in multivariable regression analysis
